# Reconstruction of Radiated Gluteal Defects following Sarcoma Resection with Pedicled Sensate Tensor Fascia Lata Flaps

**DOI:** 10.1155/2015/971037

**Published:** 2015-08-03

**Authors:** Albert H. Chao, Patrick N. Kearns

**Affiliations:** Department of Plastic Surgery, The Ohio State University, Columbus, OH 43212, USA

## Abstract

Sarcomas of the gluteal region often result in sizable defects following resection that are challenging to reconstruct due to their location, particularly in patients who have received radiation therapy. Reconstruction of these defects has been seldom discussed in the literature. We present two patients with large radiated gluteal defects following sarcoma resection, of which one patient received neoadjuvant radiation and the other received intraoperative radiation therapy. As a result of the resection and radiation, local tissues and recipient vessels were unsuitable for use in reconstruction. A pedicled tensor fascia lata (TFL) flap was therefore performed in both cases, which resulted in durable sensate reconstruction with good functional outcomes and no complications. We believe the pedicled TFL flap represents an important option for the reconstruction of oncologic gluteal defects that provides well-vascularized and sensate tissue from outside the zone of radiation without the need for microsurgical techniques.

## 1. Introduction

Reconstruction of large gluteal soft tissue defects has been seldom discussed in the literature, in contrast to sacral and ischial defects. The gluteal region can be challenging to reconstruct due to the paucity of local flap options and posterior location as a site of pressure and shear. Previously described techniques involving local flaps are limited and can be particularly restricted in cancer patients such as sarcoma patients, where preoperative or intraoperative radiation therapy may compromise adjacent tissues [1]. Free flap reconstruction is an alternative but can be complicated by limited recipient vessels and demanding postoperative care [2, 3].

The pedicled tensor fascia lata (TFL) flap has most commonly been utilized for reconstruction of trochanteric pressure sores and lower abdominal wall defects [4–6]. To our knowledge, the TFL flap has not been previously described for use in the reconstruction of extensive oncologic gluteal defects. In this paper, we present 2 cases of such defects reconstructed with sensate pedicled TFL flaps after sarcoma resection. The TFL flap has several potential advantages in this patient population: (1) provision of a large amount of vascularized tissue with an axial blood supply as a pedicled flap, (2) location outside the zone of radiation therapy in previously radiated patients, (3) preservation of sensation, and (4) minimal donor site morbidity.

## 2. Case Report

### 2.1. Case 1

**Figure 1:**
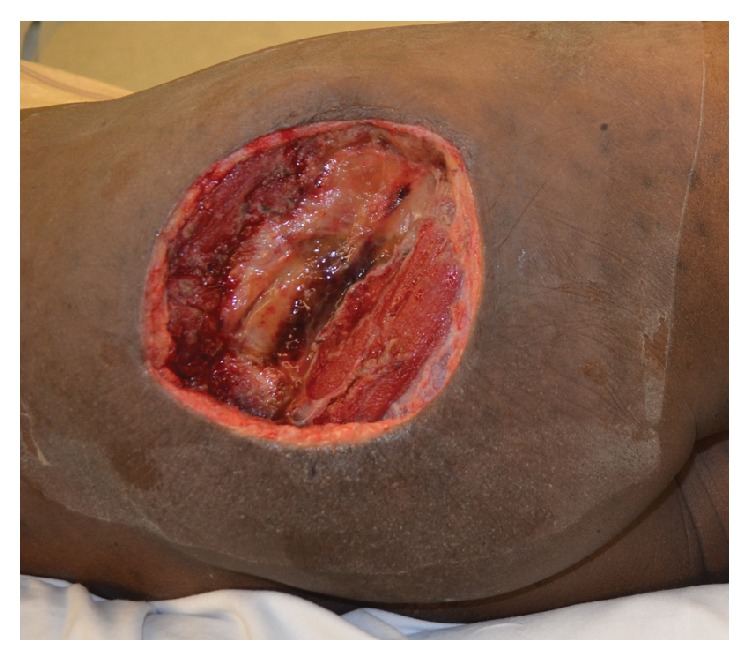
A 15 × 15 cm right gluteal defect following resection of a high-grade pleomorphic undifferentiated sarcoma following neoadjuvant radiation therapy.

**Figure 2:**
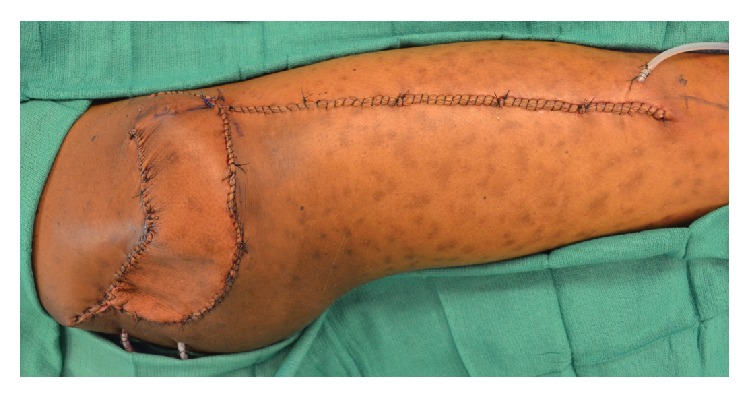
Immediate postoperative result following gluteal reconstruction with a pedicled tensor fasciae lata flap.

A 52-year-old male and active smoker presented with progressive right gluteal swelling and discomfort for several months. An ultrasound study was performed that demonstrated a fluid collection, aspiration of which showed atypical cells suspicious for malignancy. Subsequent core biopsy revealed high-grade pleomorphic undifferentiated sarcoma. After multidisciplinary evaluation, the plan for this patient was neoadjuvant chemotherapy and radiation therapy, followed by surgical resection and reconstruction. The patient proceeded to receive doxorubicin, ifosfamide, and 50 Gy of external beam radiation therapy. Approximately 8 weeks later, the patient underwent tumor extirpation, which involved resection of skin, subcutaneous tissue, and gluteal musculature, resulting in a 15 × 15 cm defect (Figure 1). The defect was dressed with a negative pressure wound therapy dressing while awaiting final pathologic margins, which were ultimately negative. Due to prior radiation therapy, local tissues were deemed unsuitable for reconstruction, and local recipient vessels for microvascular free tissue transfer were of poor quality and of insufficient length due to the prior resection. Due to these factors and the patient's history of active tobacco use, a pedicled TFL flap was performed (Figure 2). The donor site was closed primarily. The patient was ambulatory without assistance and was discharged by postoperative day 3 and healed without complication after a follow-up of 2.5 months.

### 2.2. Case 2

A 59-year-old male presented after undergoing an excisional biopsy of an enlarging right gluteal mass at another institution, which revealed a high-grade undifferentiated pleomorphic sarcoma with positive margins. Following further multidisciplinary evaluation, a plan was formulated involving surgical resection combined with intraoperative radiation therapy (IORT), followed by staged reconstruction in order to ensure negative margins prior to flap transfer. Tumor ablation resulted in a 22 × 17 cm defect spanning from the posterior aspect of the greater trochanter to the sacrotuberous ligament and included resection of the gluteus maximus muscle, thereby exposing the sciatic nerve. This was followed by delivery of 1000 cGy with 6 MeV electrons of IORT. Final pathologic margins were negative. Due to the dimensions of the defect, free microvascular flap reconstruction was planned. However, at the time of reconstruction, significant acute radiation injury was identified in the gluteal vessels and they were of insufficient length due to the prior resection, which rendered them unsuitable as recipient vessels. A pedicled TFL flap was therefore performed, rather than a more complicated free tissue transfer procedure involving vein grafts. The donor site was closed primarily. The patient was ambulatory without assistance and was discharged by postoperative day 3 and healed without complication.

### 2.3. Anatomy and Surgical Technique

The anatomy and surgical technique of the TFL flap have been previously well described [4–6]. In brief, the TFL muscle is a thin, band-like muscle located in the lateral thigh that functions as an accessory muscle to help flex and abduct the thigh and is expendable for the purposes of soft tissue reconstruction. It originates from the anterior iliac crest, anterior superior iliac spine (ASIS), and greater trochanter, ultimately inserting onto the lateral tibial condyle. The TFL flap receives its dominant blood supply from the lateral circumflex femoral artery (LCFA), which enters the muscle along its deep aspect approximately 7–12 cm distal to the ASIS, and supplies myocutaneous and septocutaneous perforators to the skin. The sensory innervation of the TFL flap consists of the lateral cutaneous branch of T12 that enters the lateral thigh region after crossing the iliac crest approximately 6 cm posterior to the ASIS and by the lateral femoral cutaneous nerve that enters the anterior border of the lateral thigh skin 10 cm distal to the origin of the tensor fasciae lata muscle.

**Figure 3:**
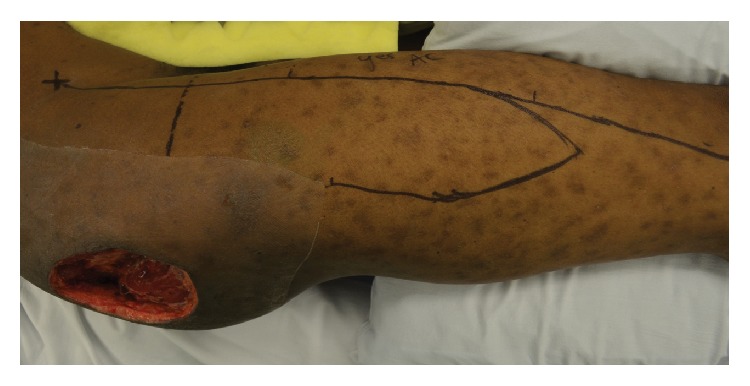
Design of a tensor fascia lata flap.

Flap elevation is performed from a lateral decubitus position. The ASIS and lateral tibial condyle are marked, as is a line adjoining these two points, which approximates the anterior border of the TFL. The flap is designed based on the dimensions of the defect, preferably along the proximal two-thirds of the thigh where perfusion is more reliable (Figure 3). Dissection of the flap begins distally, where the TFL is identified and disinserted, and then it is raised from inferior to superior. In the proximal thigh, great care is exercised to identify and preserve the vascular pedicle along the deep aspect of the flap. Further proximal flap and pedicle dissection are performed to adequately mobilize the flap. Sensory innervation to the flap is maintained by preserving both the lateral cutaneous branch of T12 and the lateral femoral cutaneous nerve. The lateral cutaneous branch of T12 is located subcutaneously 6 cm posterior to the ASIS and is thus proximal relative to flap dissection and is easily maintained during standard harvest. The lateral femoral cutaneous nerve is located subcutaneously 10 cm inferior to the ASIS and can be identified when dissecting through the subcutaneous tissues of the anterior flap incision, where it is identified and mobilized, if necessary. The intervening skin bridge between the donor and recipient sites is divided in order to prevent constriction of the flap beneath a subcutaneous tunnel. In addition, this allows for rotation and advancement of the posterior thigh tissues that facilitates donor site closure and also reduces the size of the defect (Figure 2). Closed suction drains are placed, and primary donor site closure can typically be accomplished when the flap is less than 9 cm wide; otherwise split-thickness skin grafting may be necessary.

### 2.4. Postoperative Care

In the immediate postoperative period, patients are placed on bed rest for 2 days on a low air loss mattress, with no pressure allowed on the side of flap reconstruction. On postoperative day 3, patients are permitted to stand, first with the assistance of a physical therapist, and then proceed to ambulation as tolerated. No direct pressure is permitted on the flap site for 1 month, after which patients are permitted to begin sitting on the flap for short periods of time and gradually increase their activities. Patients are permitted to return to full activities at 6 weeks.

## 3. Discussion

Most prior descriptions of techniques to reconstruct the gluteal region have focused on patients with pressure sores for whom defects are typically located over bony prominences (ischium and sacrum) and for whom there are typically suitable local flap options for soft tissue reconstruction [7]. Patients with gluteal sarcomas present a significantly different clinical scenario. In this population, large resection and prior radiation therapy often prohibit the use of local flap options. Although in these cases microsurgical reconstruction may be considered, these two factors also often render the gluteal vessels (the only recipient vessels in proximity) unsuitable for free tissue transfer, thereby necessitating a significantly more complicated procedure involving vein grafts and position changes during surgery [2, 3]. The anterolateral thigh (ALT) flap represents a pedicled option for reconstruction of gluteal defects; however, this is often limited to lateral defects since it is located further from the buttocks than the TFL flap [8, 9]. In addition, in our experience, the TFL flap is more easily harvested from a lateral decubitus position compared to the ALT flap, to allow for simultaneous access to the gluteal defect.

Although the TFL flap has been well described in the reconstruction of trochanteric pressure sores and abdominal wall defects, to our knowledge it has not been previously described for the reconstruction of gluteal defects. In this report, we describe reconstruction of two cases of large gluteal defects following sarcoma resection. In both patients, a free tissue transfer was considered but was complicated by the nature of the resection and prior radiation therapy and, although feasible, would have necessitated a more complex procedure and postoperative course. Instead, reconstruction was successfully achieved with pedicled sensory TFL flaps, with minimal morbidity.

## 4. Conclusions

Extensive gluteal defects can be reconstructed with the pedicled TFL flap, which allows for transfer of a large amount of well-vascularized tissue, as well as primary donor site closure, minimal donor site morbidity, and sensate reconstruction. We believe that the pedicled TFL flap represents a useful option in patients with oncologic gluteal defects, particularly if prior radiation therapy has compromised local tissues and recipient vessels.
